# Risk factors of early mortality within 72 hours of birth in neonates born at 22 23 weeks' gestation

**DOI:** 10.1186/s12887-025-06311-2

**Published:** 2025-12-07

**Authors:** Tomonori Kurimoto, Takuya Tokuhisa, Asataro Yara, Hiroshi Ohashi, Eiji Hirakawa, Takatsugu Maeda, Masato Kamitomo

**Affiliations:** Department of Neonatology, Perinatal Medical Center, Kagoshima City Hospital, 37-1 Uearata, Kagoshima, Japan

**Keywords:** Mortality rate, Apgar score, Early death, Fetal bradycardia, Neonates, Survival-to-discharge rate

## Abstract

**Background:**

The survival rates of neonates are significantly influenced by gestational age, with further differences observed internationally. Survival rates for live births at 22 and 23 weeks of gestation range from 3.7% to 56.7% and from 20.0% to 79.3%, respectively. Despite advancements in neonatal care, the mortality rate in these preterm infants remains high, and the factors influencing mortality remain unclear. In this study, we conducted a comparative analysis of the risk of death within 72 h of birth in neonates born at 22 and 23 weeks' gestation.

**Methods:**

This single-center, retrospective study analyzed 185 patients admitted to the neonatal intensive care unit between January 2006 and December 2023. Maternal information, placental pathology, out-of-hospital births, and neonatal information were compared between patients who did and did not succumb to mortality within 72 h. A logistic regression model was created with death within 72 h as the outcome variable, and fetal bradycardia, 5-min Apgar score, Umbilical arterial pH (UA pH), and tension pneumothorax as explanatory variables. Parameter estimation was performed using the likelihood method.

**Results:**

In the death within 72 h after birth group, differences were observed in the mode of delivery determined by fetal bradycardia (4/15 cases, 26.7%), 1-min Apgar score (1, 95% confidence interval [CI]: 1–3 points), 5-min Apgar score (5, 95% CI: 2–6 points), UA pH (7.24, 95% CI: 7.11–7.32), and tension pneumothorax (7/15 cases, 46.7%). In the logistic regression model estimating risk factors of death within 72 h, the expected values of the regression coefficients and 95% CI were as follows: fetal bradycardia (7.89, 95% CI: 1.31–48.40), 5-min Apgar score (1.43, 95% CI: 1.04–2.03), UA pH (6.10, 95% CI: 0.09–300.93), and tension pneumothorax (8.79, 95% CI: 2.35–35.46).

**Conclusion:**

Death within 72 h of birth in neonates born at 22–23 weeks gestation is associated with fetal bradycardia, low 5-min Apgar scores, and tension pneumothorax. Optimizing prenatal care, timely resuscitation, and neonatal management strategies may improve survival outcomes.

## Background

Neonatal survival rates can vary considerably depending on the gestational age at birth, with differences further observed internationally. Survival rates for live births at 22 weeks of gestation range from 3.7% to 56.7%, while those at 23 weeks of gestation range from 20.0% to 79.3% [1]. Recent studies have identified advancements in neonatal care practices, particularly in the provision of proactive care for infants born at 22 weeks’ gestation. For example, one population-based cohort study in England and Wales highlighted a three-fold increase in the provision of survival-focused care from 2018 to 19 to 2020-21, accompanied by increases in neonatal unit admissions and survival-to-discharge rates [2].

Prior systematic reviews and meta-analyses have further explored the outcomes of proactive neonatal treatment at 22 weeks of gestation, indicating an overall survival rate of 29.0% in studies spanning 2000–2020, with a significant upward trend over the years [3]. Historical data from Japan’s Neonatal Research Network between 2003 and 2012 underscored the improving survival rates in infants born at 22 and 23 weeks of gestation, reaching as high as 46.1% and 72.9%, respectively [4]. The survival-to-discharge rates of infants following active resuscitation in our Neonatal Intensive Care Unit (NICU) from 2006 to 2015 were 75% and 62% at 22 and 23 weeks of gestation, respectively [5]. However, despite proactive care, the mortality rate within the first 72 h of life was still 8.1% (8/99 infants).

Despite these advancements, challenges persist, particularly those highlighted by high early mortality rates within the first 72 h of life, even with proactive approaches. Understanding the factors contributing to mortality in neonates born at 22–23 weeks’ gestation is therefore crucial for refining the strategies aimed at reducing morbidity and improving outcomes in this vulnerable population.

The present study aimed to analyze the risk factors associated with early mortality among neonates born at 22–23 weeks of gestation over 18 years (2006–2023), contributing to ongoing efforts to enhance prenatal and postnatal care practices.

## Methods

### Study design and study participants

This single-center retrospective cohort study focused on neonates born at 22 weeks and 0 days to 23 weeks and 6 days of gestation between January 1, 2006, and December 31, 2023, who were actively resuscitated and admitted to the NICU of Kagoshima City Hospital. Both inborn neonates and those transferred after delivery at outside facilities were included in the study. This tertiary medical center in Kagoshima Prefecture manages both maternal and neonatal transfers from primary obstetric institutions. During the study period, many mothers with a high risk of delivery at 22–23 weeks of gestation were transferred to our hospital. For those who could not be transferred when delivery was imminent, our neonatologist rapidly attended the facility to which the mother was admitted, arriving at the time of delivery or shortly thereafter to perform resuscitation. After stabilizing the newborn’s condition, the neonates were transferred to our hospital in a dedicated neonatal transport vehicle, accompanied by the neonatologist. Depending on the maternal and fetal conditions, neonates were delivered by cesarean section or vaginal delivery at 22–23 weeks of gestation. Neonatal resuscitation was not restricted based on gestational age or birth weight. Active resuscitation included continuous positive airway pressure, endotracheal intubation, and surfactant replacement therapy, and was provided to all neonates. Data were collected from the electronic medical records. Gestational age was calculated based on the last menstrual period, crown-rump length using early pregnancy ultrasound data, or both. Antepartum stillbirth was defined as fetal death before labor, and intrapartum stillbirth as fetal death during labor. Between 2006 and 2023, 187 neonates were delivered at 22–23 weeks of gestation, among which there were 20 stillbirths (9 at 22 weeks and 11 at 23 weeks). During this period, all neonates born at 22–23 weeks’ gestation required resuscitation. Finally, two neonates with congenital heart disease (Tetralogy of Fallot or critical pulmonary stenosis) were excluded from the analysis. Maternal information, placental pathology, fetal heart rate monitoring, and neonatal information were compared between 15 patients (8.1%) who died within 72 h and the 170 patients (91.9%) who survived.

### Management and outcomes

The antenatal steroids (ANS) course was defined as the administration of at least two doses of ANS before delivery during the current pregnancy to promote fetal lung maturation [6].

Abnormal fetal heart rate (FHR) was assessed according to the 2009 recommendations of the National Institute of Child Health and Human Development. We evaluated late decelerations (recurrent, occurring in ≥ 50% of uterine contractions; persistent, occurring in all uterine contractions) and bradycardia (FHR < 110 bpm for ≥ 10 min) [7, 8].

The pathological placental findings included chorioamnionitis and funisitis, staged according to the Blanc classification [9]. Bacteremia was defined as the detection of microorganisms in blood cultures. Head and cardiac ultrasound examinations were performed by neonatologists within 24 h of birth, three to four times on day one, three times on days two and three, and two to three times on day four. Cardiovascular support, including dopamine, dobutamine, or steroids, was provided based on vital signs and echocardiographic assessments. The timing of indomethacin or ibuprofen administration to close a patent ductus arteriosus (PDA) in symptomatic patients and follow-up were assessed using echocardiography [10].

The diagnosis of intraventricular hemorrhage (IVH) was based on Papile’s grading system (grades Ⅰ-Ⅳ), assessed by cranial ultrasonography [11, 12]. Meconium-related ileus was diagnosed based on histopathological examination [13].

Tension pneumothorax was diagnosed based on radiographic findings of lung displacement away from the chest wall, diaphragm depression, contralateral mediastinal shift, or radiolucency along the posterior axillary line on the suspected side, and treated with needle aspiration and chest tube insertion. Neonates with birth weights below the 10th percentile were evaluated based on the standardized Japanese birth size data.

All neonates were administered empiric broad- spectrum antibiotics (ampicillin and gentamicin) for 5–7 days after birth, together with antifungal therapy using micafungin, which was continued until the humidification level decreased below 80%.wagents at birth.

Oral miconazole was administered at birth to prevent the development of necrotizing enterocolitis (NEC) for 21 days after birth [14].

Resuscitation, insertion of arterial and venous lines after birth, fluid volume, electrolyte balance, nutrition, humidity, and temperature control in the incubator were performed according to our hospital’s previous report [15].

### Statistical analyses

Comparisons between the two groups were performed using the chi-square or Fisher’s exact tests, with a significance level set at *P* < 0.05, and a 95% confidence interval (CI). Mortality within 72 h was set as the outcome variable. Variables with *P* < 0.05 in the univariate analysis (fetal bradycardia, 5-min Apgar score, umbilical artery pH, and tension pneumothorax) were entered into the multivariate logistic regression model. The selection of variables was based on both statistical significance and clinical relevance. Because only 15 deaths occurred within 72 h, the number of variables was restricted to avoid model overfitting. A logistic regression model was constructed, and parameter estimation was conducted using the maximum likelihood method. Statistical analyses were performed using R software (R Software for Statistical Computing, Vienna, Austria). (SAS Institute Inc., Cary, NC, USA).

## Results

### Clinical management

ANS therapy was administered to 67 neonates (38.4%). Cesarean delivery was required in 126 patients (68.1%). During resuscitation in our delivery room, all neonates (100%) were intubated and received surfactant replacement therapy in the delivery room or NICU.

Among transferred neonates, all 16 (100%) were intubated and given surfactant at the referring hospital prior to transport.

Upon admission to the NICU, synchronized intermittent mandatory ventilation was applied for 185 cases, with 13 infants (7%) immediately switched to high-frequency oscillatory ventilation with hypoxemia and hypercapnia. Empiric antibiotic and antifungal therapies were started in all neonates (100%) following vascular line insertion. Phenobarbital was administered as a sedative in 39 patients (21.2%), while fentanyl was used as a sedative in 55 patients (29.7%). Pharmacologic closure of patent ductus arteriosus (PDA) was attempted in 138 infants (74.6%) within the first 72 h after birth, primarily using intravenous.

indomethacin or ibuprofen according to standard neonatal intensive care protocols [10]. Treatment was discontinued once echocardiography confirmed ductal closure.

### In-hospital morbidity and mortality

The in-hospital outcomes of enrolled neonates are presented in Table 1. There were 15 deaths (8.1%) within the first 72 h of life and 170 survivors (91.9%) after 72 h. Severe IVH grade 3 or higher occurred in 6 patients (40%) in the death group and 49 (28.8%) in the survival group within the first 72 h. The present study included 10 cases of fetal bradycardia at birth. Of these, one involved an out-of-hospital birth at a primary facility, culminating in neonatal transport due to fetal bradycardia from placental abruption and subsequent cesarean delivery. At this patient’s birth, the neonatologist had not yet arrived; hence, the available nurses and obstetricians administered oxygen, performed bag-mask ventilation, and conducted chest compressions; however, bradycardia persisted. After the neonatologist arrived and initiated intubation, bradycardia improved. The neonate was transferred to our NICU, diagnosed with grade 4 IVH upon admission, and died within 72 h. Of the three cases in which the mother was urgently transported and admitted for fetal bradycardia, one involved vaginal delivery with the amniotic sac protruding into the vagina, resulting in grade 3 IVH upon admission. Another patient had a footling breech in which the fetus’s leg protruded, necessitating an emergency cesarean delivery, with grade 2 IVH upon admission. The third case involved continuous fetal bradycardia, requiring emergency cesarean delivery, with grade 3 IVH upon admission; however, the infant died within 72 h. Six cases involved emergency maternal transport and inpatient management leading to fetal bradycardia. One case in which a cesarean delivery was not desired culminated in a vaginal breech delivery with grade 4 IVH upon admission. Another case involved preterm premature rupture of membranes (PPROM) one week prior, resulting in vaginal delivery with bradycardia and subsequent death despite steroid, catecholamine, and transfusion administration. The third patient was born via emergency cesarean section due to placental abruption after the mother had experienced PPROM 2 weeks prior. Upon admission, the neonate was diagnosed with grade 2 IVH. Another case of fetal growth restriction (FGR) (−3.0 standard deviation) and fetal bradycardia necessitate a cesarean delivery for fetal indications, presenting with grade 4 IVH upon admission. One case involved placental abruption and an emergency cesarean delivery, resulting in grade 4 IVH upon admission. In the final case, the mother developed PPROM, which was followed by fetal bradycardia, necessitating an emergency cesarean delivery. Upon admission, the neonate was diagnosed with grade 3 IVH, and died shortly after birth, despite the administration of steroids, catecholamines, and blood transfusion. Among the ten neonates with fetal bradycardia, 6 (60%) survived for 72 h; however, only 2 (20%) were discharged alive.

**Table 1:** Characteristics and in-hospital outcomes of neonates born at 22–23 weeks’ gestation

	Death (% or IQR)	Survival (% or IQR)	*p*-value
Variables	15 (8.1)	170 (91.9)	
Maternal age (years)	30 (27–35)	31 (27–34)	0.39
Primipara	5 (33.3)	96 (56.5)	0.11
C/S	12 (80.0)	114 (67.1)	0.25
P/D	3 (20.0)	10 (5.9)	0.08
Severe P/D	1 (6.7)	5 (2.9)	0.4
V/D	5 (33.3)	34 (20.0)	0.32
L/D	2 (13.3)	21 (12.4)	1
Recurrent L/D	1 (6.7)	12 (7.1)	1
Persistent L/D	1 (6.7)	3 (1.8)	0.29
Severe L/D	1 (6.7)	4 (2.4)	0.34
Fetal bradycardia	4 (26.7)	6 (3.5)	0.005
Fetal tachycardia	0	5 (2.7)	1
Tocolysis	8 (53.3)	117 (68.8)	0.25
MgSO4	6 (40.0)	94 (55.3)	0.29
Ritodrine	10 (66.7)	115 (67.6)	1
Antibiotics	5 (33.3)	59 (34.7)	1
PROM	8 (53.3)	55 (32.4)	0.15
CAM stage 2–3	10 (66.7)	84 (49.4)	0.28
Funisitis stage 2–3	6 (40.0)	39 (22.9)	0.2
HDP	1 (6.7)	4 (2.4)	0.35
Smoking	0	2 (1.2)	1
GDM	0	4 (2.4)	1
HELLP	0	3 (1.8)	1
Placenta previa	0	2 (1.2)	1
Abruption	3 (20.0)	13 (7.6)	0.1
Fertility treatment	1 (6.7)	20 (11.8)	1
Cephalic presentation	8 (53.3)	107 (62.9)	0.58
Breech presentation	7 (46.7)	52 (30.6)	0.25
Transverse lie	0	3 (1.8)	1
Footling breech presentation	0	6 (3.5)	1
ANS	4 (26.7)	67 (39.4)	0.41
Single dose of ANS	0	21 (12.4)	0.22
One course of ANS	4 (26.7)	46 (27.1)	1
Cervical cerclage	1 (6.7)	11 (6.5)	1
Admission-day delivery	3 (20.0)	39 (22.9)	1
Sex (male)	7 (46.7)	89 (52.4)	0.79
22 w	2 (13.3)	55 (32.4)	0.15
Birth weight (median)	514 (471–600)	549 (476–602)	0.79
FGR (10% tile)	3 (20.0)	20 (11.8)	0.41
Out-of-hospital birth	2 (13.3)	14 (8.2)	0.62
MD twin	1 (6.7)	9 (5.3)	0.58
DD twin	0	28 (16.5)	0.13
TTTS	0	8 (4.7)	1
APS 1 min	1 (1–3)	3 (1–4)	0.03
APS 5 min	5 (2–6)	6 (5–7)	0.002
UA pH	7.24 (7.11–7.32)	7.33 (7.27–7.38)	0.007
IVH grade 3–4	6 (40.0)	49 (28.8)	0.38
EOS	2 (13.3)	9 (5.3)	0.22
TP	7 (46.7)	18 (10.6)	0.001
RDS	15 (100)	170 (100)	1
MRI	0	5 (2.9)	1

Fertility treatment refers to artificial insemination by husband, intracytoplasmic sperm injection, and in vitro fertilization and embryo transfer

*AIH* artificial insemination by husband, *ANS* antenatal steroids, *APS* Apgar score, *CAM* chorioamnionitis, *C/S* cesarean section, *DD twin* dichorionic diamniotic twin, *EOS* early onset sepsis, *FGR* fetal growth restriction, *GDM* gestational diabetes mellitus, *HELLP* Hemolysis, Elevated Liver Enzymes, and Low Platelet Count Syndrome in Pregnancy, *HDP* hypertension disorders of pregnancy, *IQR* interquartile range, *IVH* intraventricular hemorrhage, *L/D* late deceleration, *MD twin* monochorionic diamniotic twin, *MRI* meconium-related ileus, *P/D* prolonged deceleration, *PROM* premature rupture of membranes, *RDS* respiratory distress syndrome, *TP* tension pneumothorax, *TTTS* twin-to-twin transfusion syndrome, *UA pH* umbilical arterial blood gas pH, *V/D* variable deceleration

Overall, 16 neonates were born at the primary facility. Two transferred neonates (12.5%) were not attended to by a neonatologist at birth. One case involved that of an infant born outside of the hospital with fetal bradycardia, as described above. Bradycardia continued after birth, requiring chest compressions and mask ventilation for 10 min until the neonatologist arrived, after which intubation resulted in an increase in heart rate. The other case also involved bradycardia after birth, requiring chest compressions and mask ventilation until the neonatologist arrived 1 h later, intubated the infant, administered intratracheal epinephrine, and established an umbilical venous line for epinephrine administration, resulting in heart rate recovery. However, despite treatment, both neonates died within 72 h after birth following admission to our hospital.

**Fig. 1:**
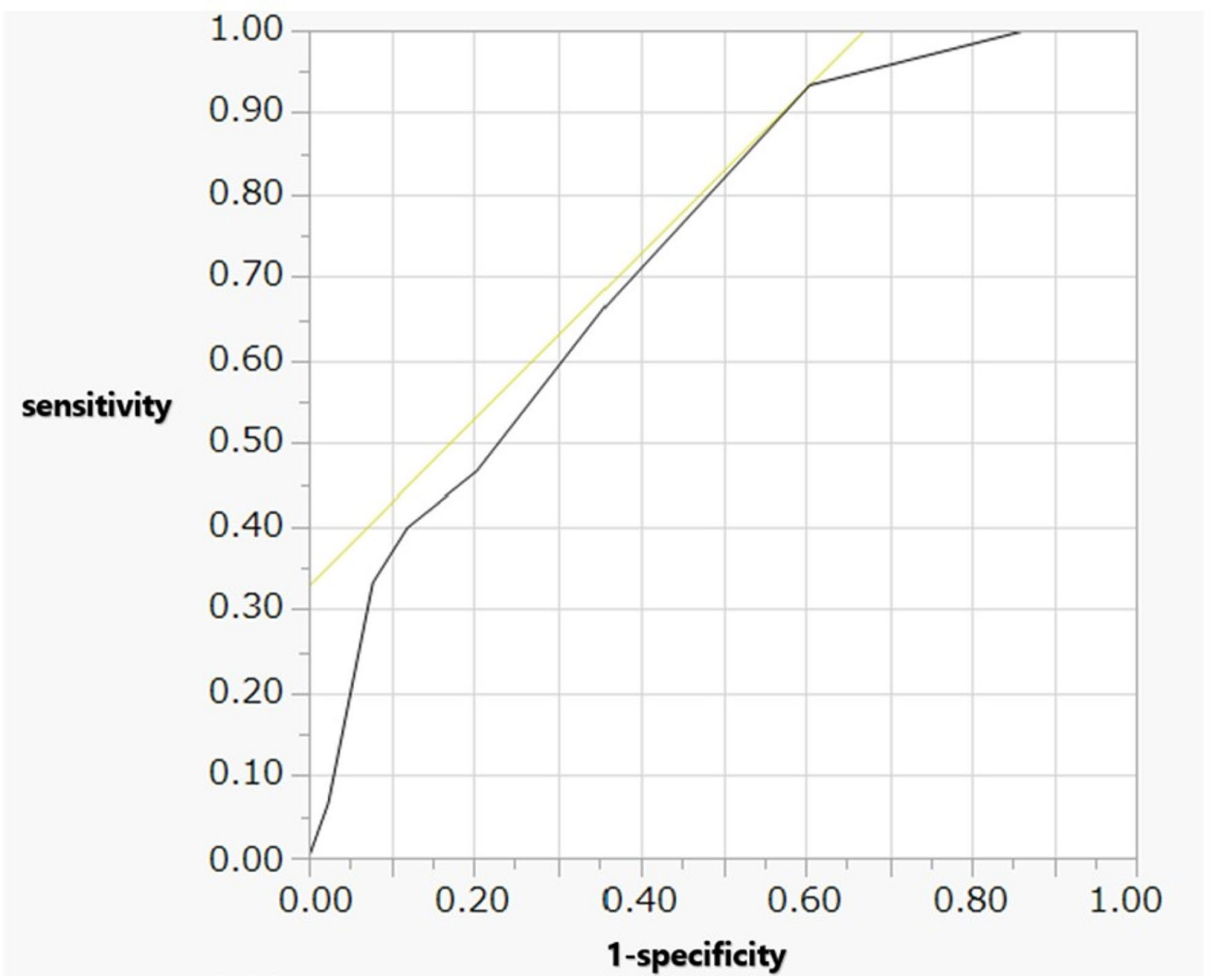
ROC curve analysis of the Apgar score at 5 min to predict mortality within 72 h of birth. When the cut-off value for the 5-min Apgar score was set at 6, an AUC of 0.73, sensitivity of 0.93, 1-specificity of 0.60, and a likelihood ratio of a positive result of 1.54 were obtained ROC, Receiver Operating Characteristic; AUC, Area Under the Curve

**Fig. 2:**
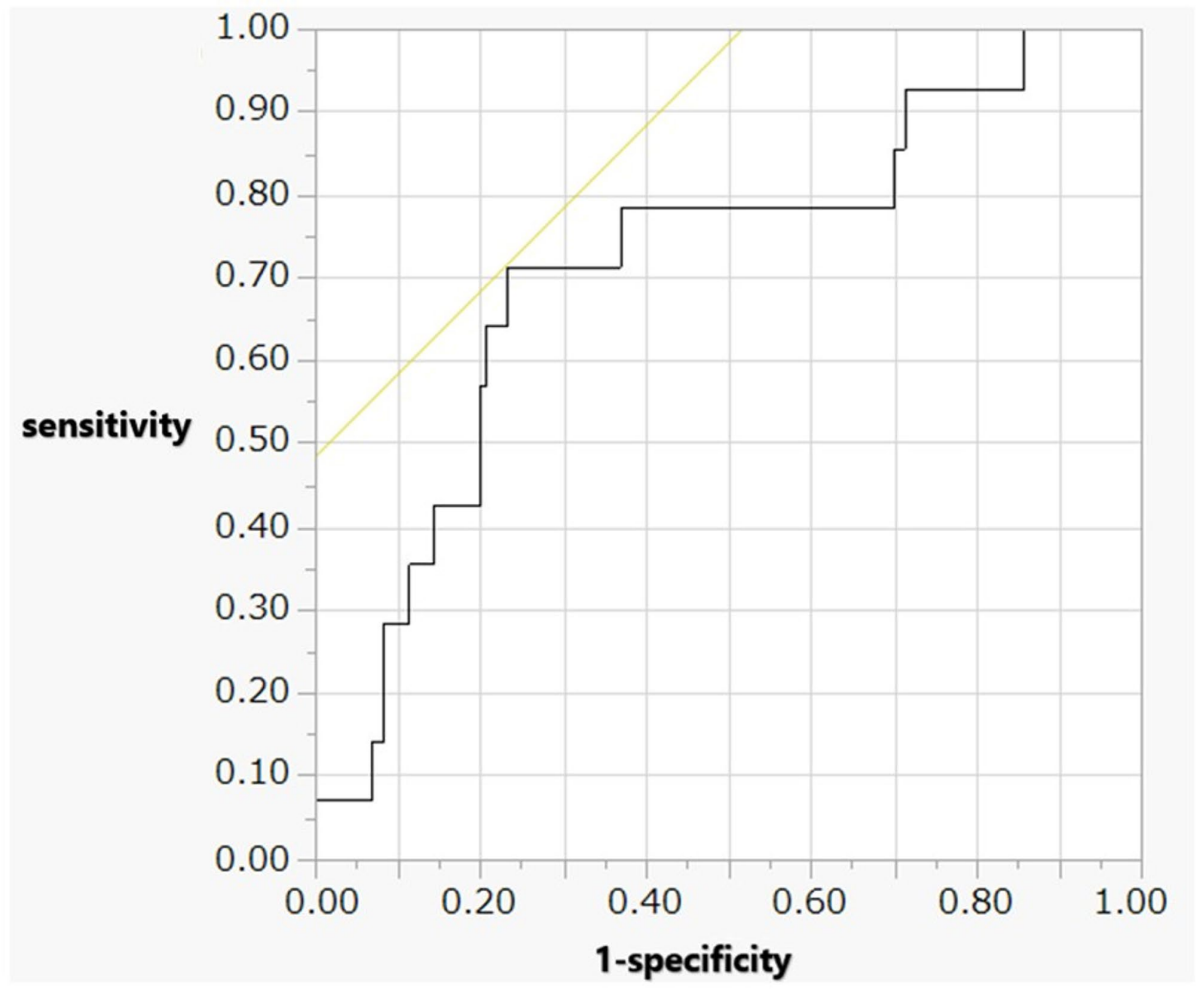
ROC curve analysis of the UA pH to predict mortality within 72 h of birth. When the cut-off value for the UA pH was set at 7.265, an AUC of 0.72, a sensitivity of 0.71, a 1-specificity of 0.23, and a likelihood ratio of a positive result of 3.09 were achieved. UA, Umbilical Arterial; ROC, Receiver Operating Characteristic; AUC, Area Under the Curve

Univariate analysis between the two groups revealed significant differences in fetal bradycardia, umbilical arterial pH, 1-minute Apgar scores, 5-minute Apgar scores, and the morbidity of tension pneumothorax. In logistic regression model with death as the dependent variable, the following odds ratio (OR) were calculated: 7.9 for fetal bradycardia (95% CI: 1.31–48.40), 6.1 for umbilical arterial pH (95% CI: 0.09–300.93), 1.4 for 5-minute Apgar score (95% CI: 1.03–2.03), and 8.8 for tension pneumothorax (95% CI: 2.35–35.46) (Table 2) The cut-off value for the 5-min Apgar score was 6, with an AUC of 0.73 and a likelihood ratio of a positive result of 1.54 (Table 3; Fig. 1). The cutoff value of UA pH was 7.265, with an AUC of 0.72 and likelihood ratio of a positive result of 3.09 (Table 3; Fig. 2).

**Table 2:** Multivariable logistic regression analysis of risk factors of mortality in neonates born at 22–23 weeks’ gestation

Logistic regression models	Odds ratio	CI (95%)
Bradycardia	7.9	1.31–48.40
UA pH	6.1	0.09–300.93
APS 5 min	1.4	1.03–2.03
Tension pneumothorax	8.8	2.35–35.46

*APS* Apgar score, *CI* confidence interval, *UA pH* umbilical arterial blood gas pH

**Table 3:** Cut-off values of APS at 5 min and UA pH levels to predict mortality in infants born at 22–23 weeks’ gestation

	Cut-off values	AUC	Sensitivity	1-specificity	LR of positive result
APS 5 min	6	0.73	0.93	0.60	1.54
UA pH	7.265	0.72	0.71	0.23	3.09

*APS* Apgar score, *AUC* area under the curve, *LR* likelihood ratio, *UA pH* umbilical arterial blood gas pH

## Discussion

The risk factors for mortality within 72 h of birth in neonates at 22–23 weeks of gestation remain largely unexplored. This observational study demonstrated that fetal bradycardia, low 5-min Apgar scores, and tension pneumothorax significantly increased the risk of mortality within 72 h after birth. Reducing these risk factors may potentially improve survival rates. Infants born via cesarean delivery at 22–23 weeks of gestation have higher survival rates (1-year survival) [16]. However, as yet, there have been no studies performing comparative analyses of fetal conditions using fetal heart rate monitoring until birth. Regarding resuscitation, 5-min Apgar scores ≤ 3 in neonates born at 22–24 weeks increase the odds of death within 24 h (OR 10.3, 95% CI: 2.33–46.0) [17]. Another study found that each 1-point increase in the 5-min Apgar score decreased the odds of death in infants born at 22–23 weeks (OR 0.62, 95% CI: 0.46–0.84) [18]. Higher Apgar scores were associated with better survival rates in infants born at 22–24 weeks [19]. Infants delivered at tertiary facilities by cesarean section before the onset of fetal bradycardia and provided with immediate professional resuscitation may avoid severe bradycardia through timely airway management, resulting in improved 5-min Apgar scores and reduced need for postnatal chest compressions.

Common causes of death within 72 h in neonates born at 22–28 weeks include respiratory distress syndrome, with respiratory failure reported as a significant cause of death within 96 h in neonates born at 23 weeks [20, 21]. In the present study, tension pneumothorax was identified as a risk factor for mortality within 72 h in neonates born at 22–23 weeks. Air leaks result from the rupture of over-distended alveoli, potentially caused by air trapping or uneven gas distribution [22]. Air progresses along the perivascular connective tissue sheaths, ultimately resulting in pneumothorax [23]. Mechanical ventilation with high inspiratory pressures or large tidal volumes can exacerbate this risk, causing alveolar rupture and fatal tension pneumothorax. In 2021, the American College of Obstetrics and Gynecology and the Society for Maternal-Fetal Medicine also updated their guidelines to recommend consideration of ANS at 22 weeks 0 days to 23 weeks, and 6 days of gestational age [24]. Among infants born between 22 0/7 and 23 6/7 weeks’ gestation who received intensive care, those who received a complete course of ANS by 22 6/7 weeks had independently higher rates of survival and survival, without any major morbidities. These findings indicate that the use of ANS may be beneficial for patients at 22 6/7 weeks’ gestation or less when active treatment is considered [25]. However, in Japan, the rate of ANS administration remains lower than in Western countries, possibly due to concerns about maternal infection or uterine contractions, as well as the difficulty of completing the full course within 48 h before delivery. Nevertheless, ANS administration should be considered whenever delivery at 22–23 weeks is anticipated.

An important contextual factor in interpreting these findings is Japan’s neonatal care policy. In Japan, resuscitation and continuation of life-sustaining treatment for extremely preterm infants are generally pursued more proactively than in many Western countries, such as the United States. Unlike in some regions, withdrawal of intensive care is rarely practiced once initiated. As a result, mortality patterns may be influenced not only by physiological and clinical factors but also by differences in cultural and ethical approaches to neonatal care. This context should be taken into account when comparing international outcomes.

Several other perinatal variables, including maternal complications, antenatal steroid use, and place of birth (inborn vs. transferred), were evaluated but did not reach statistical significance in our cohort. Given the long study period (2006–2023), changes in perinatal management and technology may also have affected outcomes. Future multicenter studies with larger sample sizes are warranted to validate these findings and further elucidate temporal trends in neonatal care practices.

The main strengths of this study include the active resuscitation of all neonates born at 22–23 weeks of gestation, the inclusion of all eligible cases in the analysis, and the relatively large sample size compared with previous reports.

However, several limitations should be acknowledged. First, this was a retrospective, single-center cohort study, which may limit the generalizability of the findings. Second, changes in perinatal management practices and medical equipment over the 17-year study period may have influenced outcomes. Third, in two cases, neonatologists were not present at the initial delivery in primary facilities, potentially introducing variability in the infants’ initial conditions. Nevertheless, because this study included all neonates born at 22–23 weeks of gestation within a single prefecture who were admitted to our hospital, the risk of selection bias was minimized. Although retrospective and non-blinded in nature, the data were extracted from comprehensive medical records to reduce information bias and ensure data reliability.

## Conclusions

Death within 72 h of birth in neonates born at 22–23 weeks of gestation is associated with fetal bradycardia, low 5-min Apgar scores, and tension pneumothorax after birth. To further validate our results, prospective, multicenter studies involving multiple facilities are required.

## Data Availability

The datasets used and/or analysed during the current study are available from the corresponding author on reasonable request.

## Abbreviations

ANS: Antenatal Steroids
CI: Confidence Interval
FHR: Fetal Heart Rate
IVH: Intraventricular Hemorrhage
NEC: Necrotizing Enterocolitis
NICU: Neonatal Intensive Care Unit
PDA: Patent Ductus Arteriosus
PPROM: Preterm Premature Rupture of Membranes
UA pH: Umbilical Arterial pH
